# Choosing the optimal target area for repeated transcranial magnetic stimulation in treating neuropathic pain in spinal cord injury patients: a comparative analysis

**DOI:** 10.3389/fneur.2024.1370420

**Published:** 2024-03-27

**Authors:** Lihua Jin, Haonan Wang, Yifei Dong, Qian Chen, Linrong Li, Yongmei Li

**Affiliations:** ^1^Department of Rehabilitation Medicine, Second Affiliated Hospital of Kunming Medical University, Kunming, Yunnan, China; ^2^Department of Rehabilitation, Kunming Medical University, Kunming, Yunnan, China; ^3^Department of Burn and Plastic Medicine, The Fourth Medical Center of the Chinese PLA General Hospital, Beijing, Beijing, China

**Keywords:** repetitive transcranial magnetic stimulation (rTMS), neuropatic pain, non-invasive stimulation, rehabilitation, spinal cord injury

## Abstract

**Objective:**

The specific target area of repeated transcranial magnetic stimulation (rTMS) in treating neuropathic pain resulting from spinal cord injury (SCI-NP) remains uncertain.

**Methods:**

Thirty-four participants with SCI-NP were allocated into three groups, namely, the motor cortex (M1, A) group, the left dorsolateral prefrontal cortex (LDLPFC, B) group, and the control (sham stimulation, C) group. The intervention was administered totally 10 times. Outcome measures assessed pre-(T0) and post-(T1)intervention, including Numerical Rating scale (NRS), anxiety (SAS), depression (SDS), sleep quality (PSQI), brief pain inventory (BPI), and impression of change.

**Results:**

All outcomes in groups A and B significantly changed after intervention (*p* < 0.05), and the delta value (T1–T0) also significantly changed than group C (*p* < 0.05). The delta value of SDS in the group B was better than the group A, and the change of pain degree in the group B was moderately correlated with the change in PSQI (*r* = 0.575, *p* < 0.05). Both patients in the groups A and B showed significant impression of change about their received therapy (*p* < 0.05).

**Conclusion:**

Both targets are effective, but LDLPFC is more effective in reducing depression in SCI-NP. Healthcare providers might select the suitable area according to the specific attributes of their patients.

## Introduction

In 2011 (1), the International Association for the Study of Pain (IASP) revised its definition of neuropathic pain (NP), to refer to “pain resulting from damage to or disease of the somatosensory nervous system.” Based on the location of the injury, NP can be categorized into peripheral neuropathic pain (PNP) and central neuropathic pain (CNP). CNP typically occurs following spinal cord injury (SCI), referred to as SCI-NP, as well as after stroke and in cases of multiple sclerosis. The prevalence rate of SCI-NP is approximately 58%, and a significant proportion (approximately 72%) of patients who experience NP within 1 month following the spinal cord injury continue to exhibit pain symptoms even after 6 months, suggesting the persistent nature of NP over time (2, 3). Due to the restricted effectiveness of traditional pharmaceutical treatments in controlling NP, it can significantly harm patients’ physical and cognitive abilities, emotional state, sleep routines, and overall well-being (4). Consequently, offering adequate support to individuals with SCI-NP poses an ongoing challenge for healthcare professionals.

The utilization of neuroregulatory techniques has led to the discovery that repeated Transcranial Magnetic Stimulation (rTMS) can effectively alleviate neuropathic pain (5). According to the rTMS application guidelines published in 2020, the application of high frequency rTMS on the Primary Motor Cortex (M1) located on the side opposite to the pain has been strongly recommended as Level A evidence for treating NP (5). However, it is important to note that the majority of this evidence is derived from studies on rTMS for PNP, while the research evidence for CNP, which poses greater challenges in terms of treatment, remains insufficient (5).

Previous research has indicated that both M1 and left dorsolateral prefrontal cortex (LDLPFC) hold promise as therapeutic targets for addressing SCI-NP (6). However, there is a dearth of studies that have directly compared the analgesic effects produced by these two stimulus targets. Notably, Huang et al. (7) reported a protocol to examining the analgesic effect of these stimulus targets on individuals with SCI-NP, yet the study remained in the experimental phase and did not furnish conclusive evidence for the application in clinical settings.

Hence, the primary objective of this research was to conduct a comparative analysis of the effectiveness of the M1 region and LDLPFC region, in relation to SCI-NP. Additionally, the study aimed to compare the analgesic effects and the other related quality of daily activity generated by these two stimulus targets with sham stimulate.

## Methods

### Study design

The present study was carried out at a regional rehabilitation center situated within a tertiary hospital. Ethical approval for this study was obtained from a tertiary hospital which is located Southwest of China Internal Review Board. The trial was registered with the Chinese Clinical Trials Register. Before their participation, all individuals were provided with comprehensive information regarding the objectives and procedures of the study, and written informed consent was obtained from each patient. Randomization lists were generated by computer, and the participants were then randomly assigned to one of three groups, namely, the M1 group (referred to as Group A, *n* = 14), the LDLPFC group (referred to as Group B, *n* = 12), or the control group (referred to as Group C, *n* = 10). A random number table was utilized to ensure the randomization process. In total, 36 individuals were enrolled and subsequently randomized into the aforementioned groups.

### Participants

The inclusion criteria for this study consisted of individuals between the ages of 18 and 70 years who had a confirmed diagnosis of spinal cord injury (SCI) with an injury level above L1. Additionally, participants were required to have a Douleur Neuropathique 4 (DN4) score and Numerical Rating scale (NRS) score of greater than 4, as well as a total duration of pain exceeding 1 month, while maintaining a stable use of analgesic drugs. Due to the intervention include the rTMS, the exclusion criteria for this study consisted metal implants in the head, pacemakers, etc., and the history of seizure, which was accompanied by other severe pain.

### Intervention

The rTMS was delivered with a magnetic-electric stimulator (CCY-III, Wuhan Yiruide Medical Equipment Co., LTD). rTMS was performed on the contralateral hemisphere of the brain experiencing pain. A figure-eight coil was utilized to assess the resting motor threshold (RMT), while the patients assumed a standard sitting posture, maintained emotional stability, and achieved muscle relaxation. When stimulating the primary motor cortex (M1) region, the coil should be positioned tangentially to the scalp. The coil should be adjusted to attain the highest amplitude and optimal repeatability of motor evoked potentials (MEPs). A positioning cap is employed for localization. The patient is instructed to don, adjust, and align the M1 area on the positioning cap with the corresponding region on their scalp that induces finger twitching (identified as left C3 and right C4 on the cap). Subsequently, the cap is shifted approximately 5 cm anteriorly to pinpoint the LDLPFC point, typically denoted as F3 on the positioning cap. The intervention was performed once a day for 10 days in 2 weeks. The circular coils used as the intervention method. The stimulation intensity was set at 80% of the RMT, with stimulation frequency of 10 Hz, stimulation duration of 4 s, and interval of 20 s, resulting in 2,000 pulses per treatment session. The intervention was administered once daily for 10 days within a 2-week period.

The M1 region was targeted for stimulation in group A subjects, while the LDLPFC region was targeted for stimulation in group B subjects. Group C subjects received the same rTMS protocol as other groups but did not undergo real rTMS intervention; the coil was turned through 90°. Additionally, all subjects across the three groups received conventional rehabilitation therapy, including physical therapy, occupational therapy, physiotherapy, pelvic floor therapy, and other relevant interventions.

The participation in groups A and B could not increase but could decrease the dosage of analgesic drugs during this study. The participation in the group C could increase the dosage of analgesic drugs, depending on the pain intensity but under the safety range. The change in dosage and data should record in the patient diary.

### Outcome measures

The Numerical Rating scale (NRS), anxiety score (measured by the Hamilton Anxiety Scale, SAS), depression (measured by the Hamilton Depression Scale, SDS), and sleep quality (measured by the Pittsburgh Sleep Quality Index, PSQI) were documented both prior to and following intervention. The NRS score, measured on an 11-point scale, serves as a concise representation of a patient’s personal perception of pain encountered. However, in the realm of clinical research, it is crucial to ascertain the statistical significance and confidence intervals pertaining to alterations in measured scores, whether within or between groups.

The Brief Pain Inventory (BPI) (8) could allow accurate comparisons between different evaluations of the same patient and patient subgroups, it also include the content such as pain-related daily activity, emotion, or relationship with other people, and it also could seem as an indirect checklist of pain-related quality of daily life.

To ascertain notable enhancements in clinical studies, a proposed approach entailed assessing the overall progress through patients’ completion of a treatment regimen, known as the Patient Global Impression Change Scale (PGIC). Consequently, the participants were queried regarding their PGIC ratings subsequent to the conclusion of the treatment, which were subsequently documented. The lower point scale dedicated the improve after intervention but the higher point dedicated worse impression.

The participants assumed a supine position on the treatment bed, and an alcohol solution was employed to cleanse the abductor pollicis brevis, thenar muscles, and wrist regions on the non-dominant hand’s M1 hand representation area. Subsequently, electrode sheets were positioned in the prescribed order of stimulation electrode, recording electrode, and grounding electrode. Circular coils were chosen for the experimental assessment, and participants were instructed to maintain a state of relaxation throughout the testing procedure. A “hot spot” was identified and designated at a specific location, where the MEP amplitude reached or exceeded 50 microvolts in at least 5 out of 10 stimuli using the lowest stimulus intensity. This finding had implications for the subjects’ RMT.

### Statistical analysis

To compare between the baseline demographic characteristics and after intervention change of the three groups, Kruskal- Wallis test or one-way ANOVA was used according to the normality of the variables. Only SAS, SDS, and PSQI scores were parametric, and these data used one-way ANOVA and, according to the Homogeneity of Variances score, Tukey, Bonferroni, or Dunnett T3 post-hoc tests. Other non-parametric data used the Kruskal-Wallis test and post-hoc Mann–Whitney U test. To compare the within-group change pre- and post- intervention, the data used Wilcoxon Signed Rank test or Paired t-test, according to the normality of the non-parametric test. Finally, correlations were conducted between the delta value of BPI, NRS between the delta value of SAS, SDS, and PSQI used Kendall’s Correlation analysis. Our focus on the direction of the difference rather than the magnitude was driven by the exploratory nature of the target stimulation and the uncertainty surrounding potential negative effects. Consequently, the delta did not employ absolute value in our analysis. All statistical analyses were performed using SPSS version 26 (IBM, NY, United States), and the statistical significance was set as *p* < 0.05. Data were plotted using GraphPad Prism 8.0 program (GraphPad Inc., San Diego, US).

## Results

### Demographic and clinical characteristics

Thirty-six participants were enrolled but two participants enrolled in group A and group B were dropped out of the trial because of consent withdrawal. Finally, thirty-four participants aged 23–69 years (mean age, 42.26 ± 11.16 years) completed this study. In total, 13 (38.23%), 11 (32.35%), and 10 (29.41%) participants were randomly assigned to the Group A, B, and C, respectively. The demographic characteristics about the injury duration, SCI type, and SCI level did not show significant different between each group. Initially, the scores of NRS, SAS, SDS, and PSQI in the baseline did not show significant difference in between-group analysis (Table 1). Due to the short duration of intervention, the dosage of analgesic use in participants in the group C almost unchanged. Conversely, one participant in the group B recorded the reduced dosage of used pregabalin after intervention compared with baseline. No safety issues or advance events were reported during trial in each group.

**Table 1:** Demographic characteristics in each group.

Group		Group A (*n* = 13)	Group B (*n* = 11)	Group C (*n* = 10)	*p* value
Age (years)		40.7 ± 14.5	40.4 ± 8.7	46.5 ± 8.0	0.161
Duration (days)		231.92 ± 372.07	163.18 ± 119.72	154.6 ± 125.46	0.959
Gender (*n*)	Male	10	8	7	0.932
	Female	3	3	3	
Ethnicity	Han	11	8	8	0.778
	Non-Han	2	3	2	
Marital statuses (*n*)	Married	12	10	10	0.644
	Unmarried	1	1	0	
SCI type	Tetraplegia	2	3	5	0.209
	Paraplegia	11	8	5	
SCI level	Complete	3	3	2	1.000
	Uncomplete	10	8	8	
NRS score		5.62 ± 0.65	5.91 ± 1.136	5.3 ± 0.675	0.220
SAS score		51.54 ± 6.641	49.55 ± 4.435	49.9 ± 4.122	0.918
SDS score		0.55 ± 0.077	0.55 ± 0.087	0.52 ± 0.028	0.305
PSQI score		11.77 ± 3.345	11 ± 3.464	9.9 ± 1.792	0.440

Group A, Repeated Transcranial Magnetic Stimulation (rTMS) intervention in the M1 area group; Group B, rTMS intervention in the LDLPFC area group; Group C, control group. SCI, spinal cord injury.

### Change of pain degree and related quality of life

**Figure 1:**
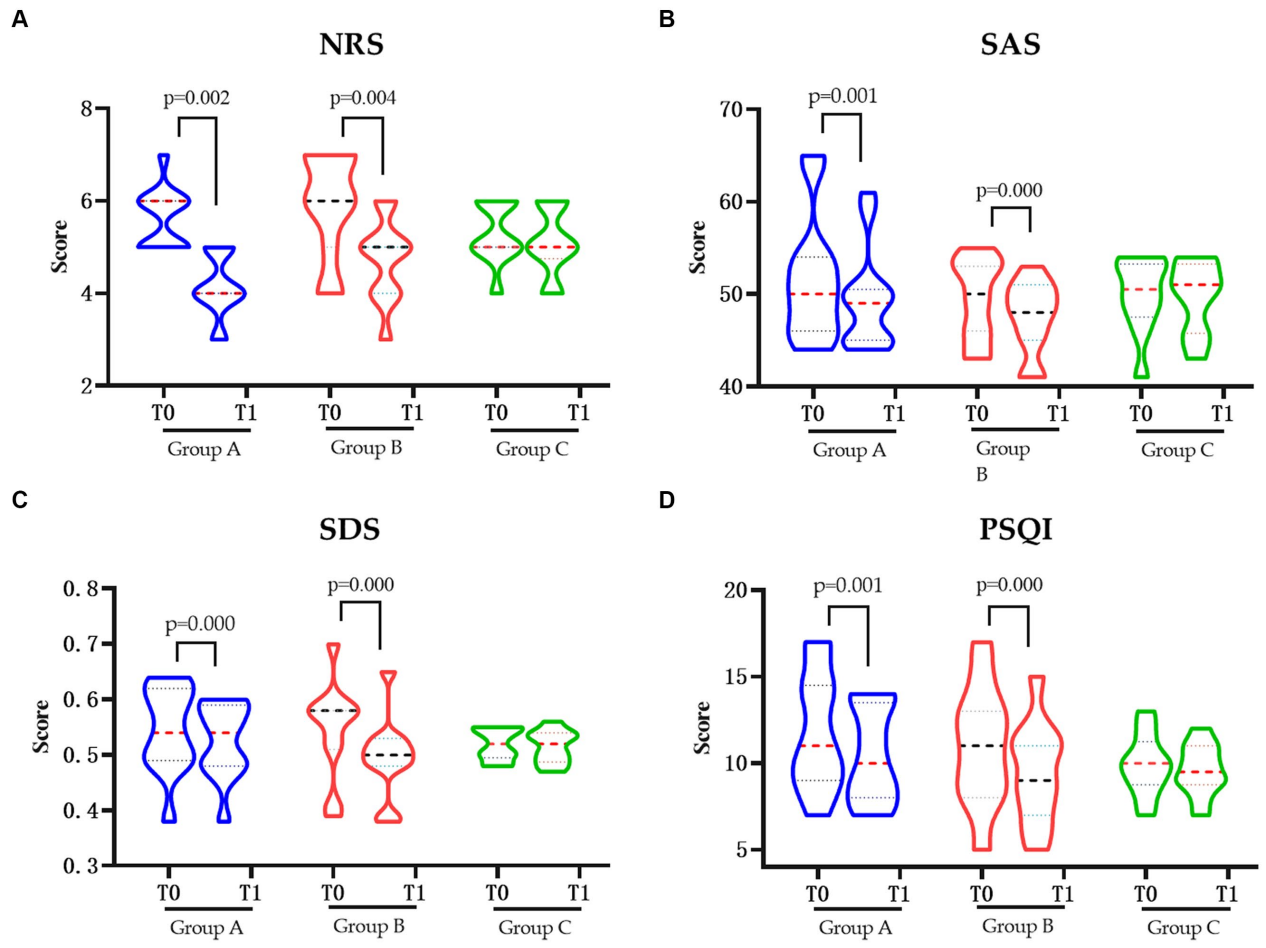
The pain and related quality of life in each group pre and post intervention. **(A)** Changes in NRS pre and post intervention. **(B)** Changes in SAS pre and post intervention. **(C)** Changes in SDS pre and post intervention. **(D)** Changes in PSQIs pre and post intervention. NRS, Numerical Rating Scale; SAS, Hamilton Anxiety Scale; SDS, Hamilton Depression Scale; PSQI, Pittsburgh Sleep Quality Index; Group A, Repeated Transcranial Magnetic Stimulation (rTMS) intervention in the M1 area group; Group B, rTMS intervention in the LDLPFC area group; Group C, the control group; SCI, spinal cord injury; T0, baseline assessment; T1, after intervention assessment.

The pain degree of NRS score was significantly decreased in groups A and B after intervention (*p* < 0.05). Similar change also showed in the SAS, SDS, and PSQI score which, regarding the anxiety, depression, and sleep quality, were also significantly decreased in groups A and B (*p* < 0.05), respectively. However, neither of changes were shown in the control group in pain degree nor the related quality of life (Figure 1).

### Change in pain-related quality of daily activity

**Figure 2:**
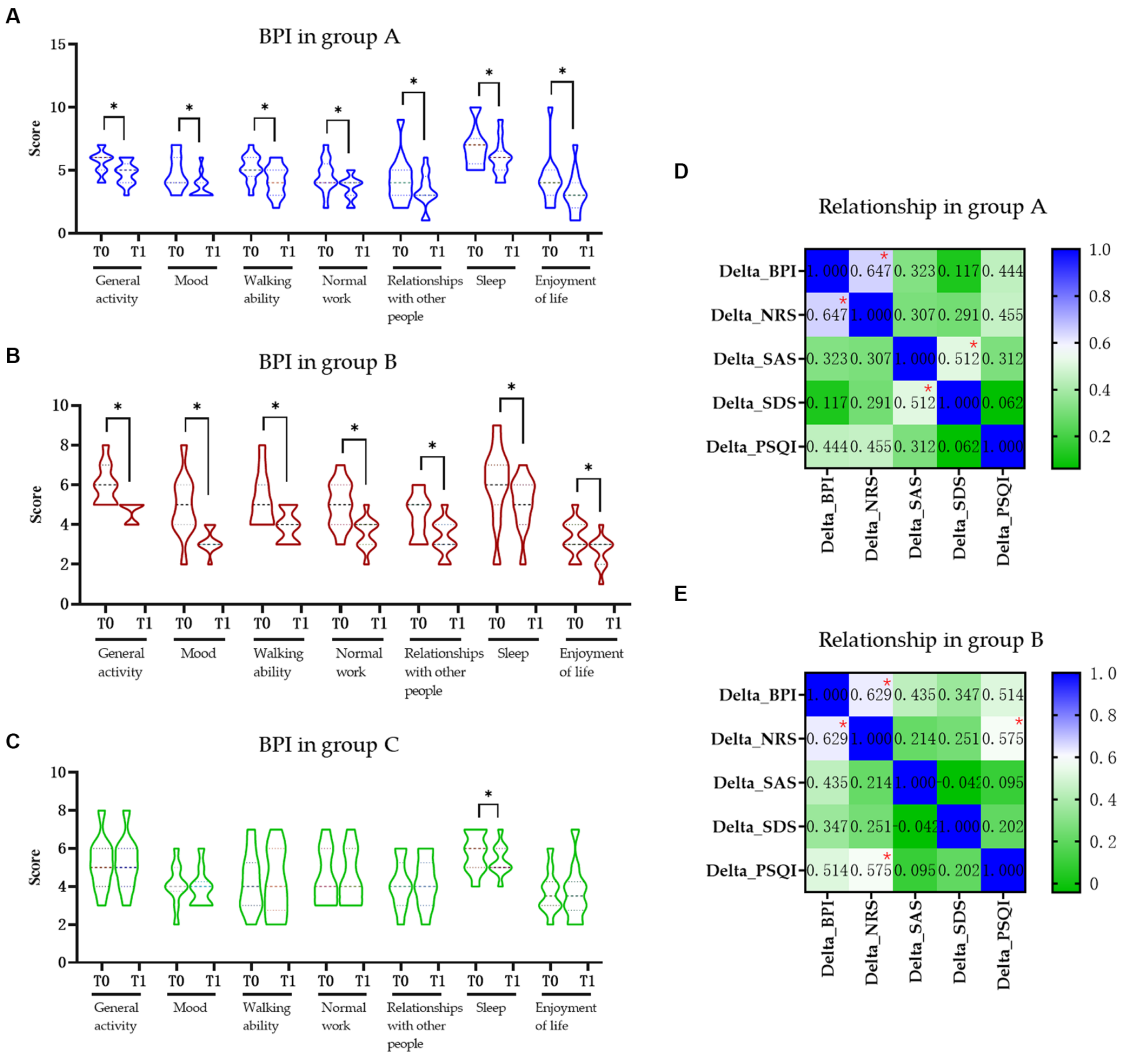
The influence of pain in daily activity and relationship of pain in life in each group. **(A)** Change in BPI score in the group A. **(B)** Change in BPI score in the group B. **(C)** Change in BPI score in the group C. **(D)** Relationship of pain and quality of life in the group A. **(E)** Relationship of pain and quality of life in group B. BPI, Brief Pain Inventory; NRS, Numerical Rating Scale; SAS, Hamilton Anxiety Scale; SDS, Hamilton Depression Scale; PSQI, Pittsburgh Sleep Quality Index; Group A, Repeated Transcranial Magnetic Stimulation (rTMS) intervention in M1 area group; Group B, rTMS intervention in LDLPFC area group; Group C, control group; SCI, spinal cord injury; T0, baseline assessment; T1, after intervention assessment; * *p* < 0.05.

All the BPI items in groups A and B were significantly decreased after intervention (*p* < 0.05) but only the “sleep” item was changed significantly in the control group (Figures 2A–C). Based on the previously established minimal clinically important difference (MCID) for the BPI, a decrease of more than 7% is considered clinically significant (9). In this study, the total BPI score in the group A was from 35.38 to 28.77 (decrease 18.70%), in the group B was from 35.09 to 26.36 (decrease 24.87%), and in the control group was from 31.60 to 31.10 (decrease 1.58%), respectively. Furthermore, when comparing the changes in delta values of the BPI, NRS, SAS, SDS, and PSQI among three groups, it was observed that while the delta value changes in groups A and B were significantly different from those in group C (*p* < 0.05), only the change in SDS in group B was significantly different from group A (*p* < 0.05) (Table 2).

**Table 2:** Changed value in each group.

	Delta BPI	Delta NRS	Delta SAS	Delta SDS	Delta PSQI
Group A	−6.62 ± 2.87	−1.46 ± 0.77	−2.15 ± 1.72	−0.02 ± 0.01	−1.30 ± 1.10
Group B	−8.87 ± 4.29	−1.18 ± 0.60	−2.00 ± 1.26	−0.05 ± 0.02^a^	−1.54 ± 0.82
Group C	−0.50 ± 1.71^a^^b^	−0.20 ± 0.42^a^^b^	−0.20 ± 1.47^a^^b^	−0.0 ± 0.01^a^^b^	−0.30 ± 0.67^a^^b^

BPI, Brief Pain Inventory; NRS, Numerical Rating Scale; SAS, Hamilton Anxiety Scale; SDS, Hamilton Depression Scale; PSQI, Pittsburgh Sleep Quality Index; Group A, Repeated Transcranial Magnetic Stimulation (rTMS) intervention in M1 area group; Group B, rTMS intervention in LDLPFC area group; Group C, control group.

a Compared with group A, *p* < 0.05.

b Compared with group B. *p* < 0.05.

### Correlation between pain and anxiety, depression, and sleep

We found moderate positive correlations of delta value of BPI between NRS in both group A (*r* = 0.647, *p* = 0.008, Figure 2D) and group B (*r* = 0.629, *p* = 0.022, Figure 2E), respectively. There also moderate positive correlation between the delta value of SAS and SDS (*r* = 0.512, *p* = 0.025; Figure 2D) in group A and delta value of NRS and PSQI (*r* = 0.575, *p* = 0.046; Figure 2E) in group B. No other significant correlations were found between other delta changes in two groups (Figure 2E).

### Impression about the intervention

**Figure 3:**
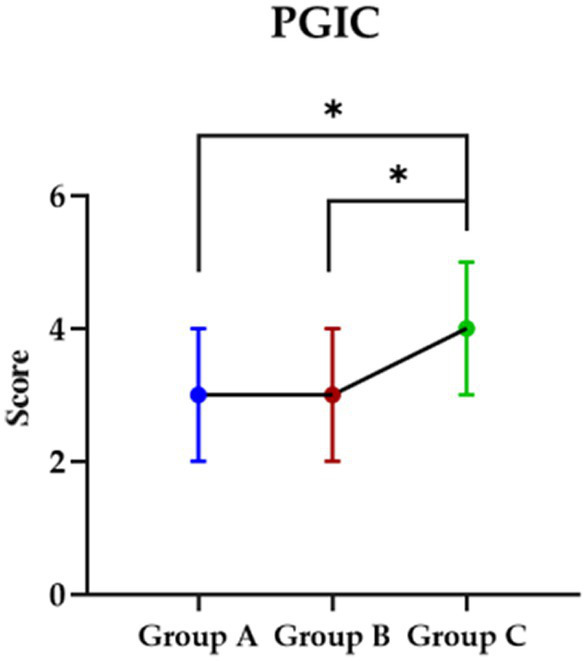
The impression in each group about they received intervention. PGIC, Patient Global Impression Change Scale; Group A, Repeated Transcranial Magnetic Stimulation (rTMS) intervention in the M1 area group; Group B, rTMS intervention in the LDLPFC area group; Group C, control group.

Post the intervention, the PGIC were checked by participants for the impression about the intervention and although both participants in group A and B showed improved than group C (*p* < 0.05), no significant was found between group A and B (*p* > 0.05) (Figure 3).

## Discussion

Spinal cord injury (SCI) is a significant affliction of the central nervous system that can result in sensory, motor, and autonomic nervous function impairment or loss. While advancements in medical technology have led to a rise in the number of individuals surviving SCI, the presence of pain as a consequence of SCI complications greatly impacts the longevity and overall well-being of these survivors. Based on statistical data, it is evident that a significant percentage of individuals with spinal cord injuries (SCI) will encounter pain, with NP being the most prevalent form of post-SCI pain. The motor and pain-related brain networks even may mutually be influenced after SCI. Moreover, the incidence of NP tends to escalate over time, thereby amplifying the likelihood of patients experiencing adverse emotional states, such as anxiety and depression. Consequently, this impedes the recovery trajectory of patients (10, 11).

Pharmacological intervention represents the prevailing therapeutic approach for the management of SCI-NP in clinical settings. However, thus far, no drug has consistently demonstrated comprehensive efficacy, predictability, safety, and suitability for long-term NP treatment (12). First-line drugs, such as gabapentin and pregabalin, as recommended internationally, only offer partial pain relief. Furthermore, research has revealed a high prevalence of abuse, approximately 70% for gabapentin and pregabalin, with pregabalin exhibiting an abuse rate exceeding 80%. Consequently, the misuse of these drugs contributes to an escalation in adverse effects (13). The aforementioned adverse effects, such as xerostomia, heightened muscular contractions, tremors, sleep disruptions, and somnolence, consequently impeding patient compliance and leading to a considerable number of patients failing to attain the desired analgesic effect or discontinuing treatment due to undesirable side effects. Therefore, there is a critical need for the advancement of innovative therapeutic strategies to mitigate the pain experienced by patients.

The suboptimal effectiveness of SCI-NP creates an opportunity for exploring alternative therapeutic interventions. Non-invasive brain stimulation (NIBS) interventions, especially rTMS, which operate on the principles of electromagnetic induction as established by Faraday, have been discovered to effectively alleviate pain (14). Its analgesic mechanism is believed to involve the activation of the descending pain control system, stimulation of brain regions associated with pain processing, and regulation of neurotransmitter expression, among other factors, ultimately resulting in pain reduction (14). Notably, rTMS offers several advantages, including affordability, painlessness, non-invasiveness, minimal side effects, high tolerance, and a strong safety profile. In a recent systematic review examining the efficacy of rTMS in the treatment of SCI-NP, eight randomized controlled trials (RCTs) were included. Among these studies, seven focused on stimulating the M1 region while one targeted the LDLPFC region. Notably, two RCTs lacked essential data. However, the quantitative analysis of the remaining six RCTs revealed that the high-frequency rTMS group experienced a significant reduction in pain intensity compared with the sham stimulation group. This finding suggests that both the M1 region and LDLPFC region may serve as effective targets for high-frequency rTMS in the treatment of SCI-NP (15).

In this study, a frequency of 10 Hz was selected for high-frequency stimulation based on two primary considerations. First, recent TMS application guidelines have designated high-frequency rTMS, targeting the contralateral M1 region as Level A evidence for the treatment of neuropathic pain (NP). Second, among frequencies such as 5, 10, and 20 Hz, 10 Hz has been commonly chosen in previous studies due to its perceived efficacy, which reported that this frequency produces satisfactory effects. The selection of intervention parameters was guided by a thorough consideration of parameters utilized in prior randomized controlled trials to ensure alignment and minimize potential discrepancies in treatment outcomes (16).

The findings of this study demonstrate that after a 10-day treatment period, regardless of the targeted stimulation region being M1 or LDLPFC, rTMS exhibits the ability to alleviate pain, improve the anxiety and depression, and improve sleep quality in patients with SCI. The general impression among SCI patients in M1 and LDLPFC groups was observed as minor improvements. Conversely, individuals who did not undergo real rTMS intervention did not experience any significant alterations in pain, anxiety, depression, or sleep quality before and after treatment. Furthermore, the degree of pain, anxiety, depression, and sleep quality improvement in both the M1 group and LDLPFC group following treatment surpassed that of the conventional treatment group. However, only the LDLPFC group exhibited a superior degree of improvement in depression compared with the M1 or control group. Although no significant disparity was observed in the analgesic effect generated by the two stimulus targets, the LDLPFC group demonstrated a greater improvement degree in pain-related inventory, as well as only the LDLPFC group showed the moderate relationship in decreased pain between related quality of daily activity. Additionally, while there is a correlation between the enhancements in pain-related and emotion-related scores in both A and B groups, it is important to note that this does not definitively establish a direct causal relationship. Emotion is influenced by numerous factors beyond pain alleviation. Nevertheless, observed reduction in pain within the LDLPFC group is also associated with improvement in sleep quality scores, indicating that this form of stimulation may enhance mood-related scores by addressing pain and enhancing sleep quality.

The potential analgesic impact of rTMS stimulation targeting the M1 or DLPFC may be linked to the engagement of the brain stem descending pain control system. Notably, when M1 was stimulated, a pronounced localized activation was observed in the thalamus, insula, cingular-orbitofrontal junction, and periaqueductal gray matter regions of the brainstem. This finding suggests a direct activation of the top-down descending pain control system which is facilitated by the functional junction between the motor corticothalamic pathway and the motor cortico-brainstem pathway (17). When the LDLPFC is stimulated, rTMS exerts widespread inhibitory effects along the descending mesencephalo-thalamic-cingulate pathway via the descending fibers originating from the prefrontal cortex (18, 19). This phenomenon is primarily attributed to the activation of anterior cingulate activity and pain control circuits, leading to the placebo effect through the release of endogenous opioids, which can be facilitated by high-frequency rTMS stimulation of the DLPFC region. Indeed, the application of rTMS stimulation to the LDLPFC region has been found to exert a significant regulatory influence on the activity of the prefrontal cortex, specifically in relation to emotional regulation and pain management in individuals diagnosed with major depression. This effect is independent of the antidepressant properties of rTMS, indicating that the LDLPFC region plays a substantial role in analgesic processes. Furthermore, the involvement of the DLPFC in attentional mechanisms and executive functioning suggests a potential connection between cognitive regulation and pain modulation. Hence, it is plausible that the application of rTMS on the LDLPFC could modulate interconnected circuits involved in mood regulation and pain perception. Consequently, the shared mechanism underlying the antidepressant and analgesic effects may be influenced by rTMS stimulation on LDLPFC. Moreover, the direct stimulation of the motor cortex can be achieved through high frequency rTMS on the M1. The extensive impact of LDLPFC stimulation not only activates the motor cortex but also regulates emotional circuits associated with pain and depression (20, 21). Moreover, utilizing a neuronavigation system to pinpoint these areas may be beneficial for more accurate feedback on stimulation outcomes.

Furthermore, previous research has substantiated the efficacy of targeting the LDLPFC region to ameliorate depressive symptoms in patients. According to the most recent rTMS application guidelines, high frequency rTMS in the LDLPFC region is classified as Class A evidence for the treatment of depression (5). All of the abovementioned evidence could explain the improvement in depression score, especially in the LDLPFC target group in the present study.

It already reported that different frequency of rTMS shows different effect that the high-frequency induces higher brain activity, whereas low-frequency rTMS suppresses cerebral cortex activity (22). In a study conducted by Zhao et al. (23), it was observed that stimulation of the M1 region using high-frequency rTMS at a frequency of 10 Hz, targeting the M1 region, and with a stimulation intensity of 90% RMT resulted in changes in cortical excitability. Following a 3-week treatment period, it was observed that the maximum amplitude of MEP significantly increased compared with pre-treatment levels, indicating an enhanced excitability of the cerebral cortex. The high-frequency of rTMS targeting the LDLPFC could improve the condition of depression (24). In the present study, we discovered that both the M1 region and LDLPFC region can serve as viable stimulus targets for high-frequency rTMS in the treatment of SCI-NP. However, the LDLPFC region exhibits greater efficacy than the M1 region in ameliorating depressive mood. It could help the clinicians to choose target area, according to the patients’ clinical characteristics.

The efficacy of interventions focused on the M1 region has been partially substantiated in the literature. For example, Ma et al. (25) demonstrated sustained pain relief for 3 months in patients who underwent 10 treatment sessions with parameters akin to those utilized in our study. Additionally, investigations employing 15 sessions of intermittent rTMS for 22 weeks have reported, enduring positive outcomes and exceeding 6 months in individuals suffering from widespread pain. Nevertheless, there is a dearth of long-term research on the effectiveness of LDLPFC-targeted therapy for SCI-NP (26). Previous studies have compared M1 and DLPFC targets, akin to our own research; however, disparities in comparison methodologies and the varying levels of pain among study participants may have impacted the results. The findings of these studies indicated that the DLPFC did not exhibit enduring analgesic effects, with immediate effects which were also comparable to those of sham stimulation. Additionally, they reported that the rTMS in DLPFC target also did not appear to reductions in depressive symptoms. Although the authors recognized the possibility that a post-hoc adjustment of the data could support the efficacy of targeting the DLPFC, no such evidence was provided (27).

## Limitation

First, the duration for recruiting patients was short, and the number of participants was restricted that the sample size was insufficient. Second, in this study, the absence of solely stimulation without oral management hindered the determination of the effect of the two targets. In fact, conducting such a study that cease oral medication and process with a sole intervention of rTMS under actual clinical conditions was challenging due to the severe pain caused by SCI-NP. The relatively brief duration of treatment in this study was a consequence of the limited length of hospital stays in clinical practice. Moreover, this constraint prevented the implementation of a cross-over trial, as a significant number of patients dropped out when the study period was extended in our pilot study. To overcome this constraint, upcoming research efforts will focus on increasing the sample size, developing stricter treatment protocols, and exploring further the underlying mechanisms by which rTMS improves SCI-NP.

## Conclusion

In this randomized study, the efficacy of two rTMS targets was assessed in order to evaluate their impact on SCI-NP. The results indicated that both of the M1 target and LDLPFC were effective, with LDLPFC demonstrating greater efficacy in reducing depression in SCI-NP. Healthcare providers might select the suitable area according to the specific attributes of their patients.

## Data availability statement

The raw data supporting the conclusions of this article will be made available by the authors, without undue reservation.

## Ethics statement

The studies involving humans were approved by Second Affiliated Hospital of Kunming Medical University Internal Review Board. The studies were conducted in accordance with the local legislation and institutional requirements. The participants provided their written informed consent to participate in this study. Written informed consent was obtained from the individual(s) for the publication of any potentially identifiable images or data included in this article.

## Author contributions

LJ: Formal analysis, Investigation, Software, Visualization, Writing – original draft. HW: Conceptualization, Data curation, Investigation, Writing – original draft. YD: Data curation, Writing – original draft. QC: Data curation, Writing – original draft. LL: Data curation, Writing – original draft. YL: Conceptualization, Funding acquisition, Writing – review & editing.
